# Supplementary material for: X‐Linked Gene Dosage and SOX2 Act as Key Roadblocks for Human Germ Cell Specification in Klinefelter Syndrome
Source: Adv Sci (Weinh). 2025 Feb 25;12(15):2410533. doi: 10.1002/advs.202410533 (PMC12005746; doi:10.1002/advs.202410533)
Supplement: Supplementary file 1 — Supporting Information [file ADVS-12-2410533-s002.pdf]

ADVANCED SCIENCE
Open Access

Supporting Information

for *Adv. Sci.*, DOI 10.1002/advs.202410533

# X-Linked Gene Dosage and SOX2 Act as Key Roadblocks for Human Germ Cell Specification in Klinefelter Syndrome

*Wenteng He, Qing Luo, Jian Zhao, Mengting Wang, Allan Zhao, Luohua Feng, Ahmed Reda, Eva Lindgren, Jan-Bernd Stukenborg, Jiayu Chen and Qiaolin Deng**

Supplemental Information

# X-Linked Gene Dosage and SOX2 Act as Key Roadblocks for Human Germ Cell Specification in Klinefelter Syndrome

Wenteng He, Qing Luo, Jian Zhao, Mengting Wang, Luohua Feng, Allan Zhao, Ahmed Reda, Eva Lindgren, Jan-Bernd Stukenborg, Jiayu Chen, Qiaolin Deng

Figure S1

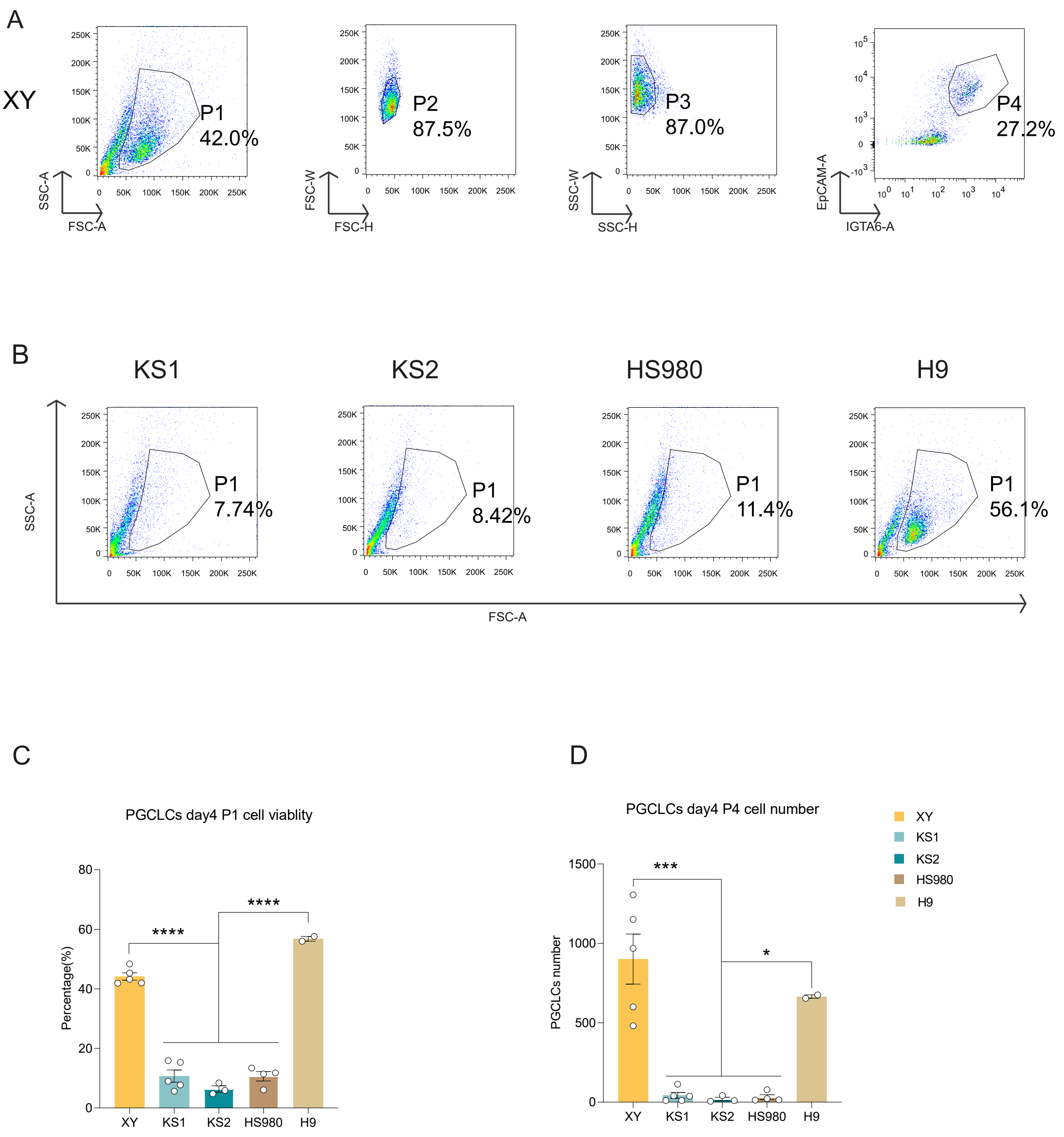

A
XY
P1
42.0%
P2
87.5%
P3
87.0%
P4
27.2%
SSC-A
FSC-A
FSC-W
FSC-H
SSC-W
SSC-H
EpCAM-A
IGTA6-A
B
KS1
KS2
HS980
H9
P1
7.74%
8.42%
11.4%
56.1%
SSC-A
FSC-A
C
PGCLCs day4 P1 cell viablity
Percentage(%)
****
XY
KS1
KS2
HS980
H9
D
PGCLCs day4 P4 cell number
PGCLCs number
***
*
XY
KS1
KS2
HS980
H9

SUPPLEMENTAL FIGURE LEGENDS

Figure S1. X-linked gene dosage underlies the differentiation efficiency of hPGCLCs, Related to Figure 1.

(A) FACS analysis of XY cells from P1 to P4. P1 represents living cell population, P2 and P3 is to gate single cell population for analysis, in the end, P4 represents the PGCLCs identified by EpCAM and IGTA6.

(B) FACS analysis of P1 cells in KS1, KS2, HS980 and H9.

(C) Statistical analysis of P1 viable cells in PGCLCs day 4 of five cell lines.

(D) Statistical analysis of P4 cell number in PGCLCs day 4 of five cell lines.

Data in (C), (D) are shown as mean ± SEM. ****$p < 0.0001$ by one-way ANOVA comparison.

Figure S2

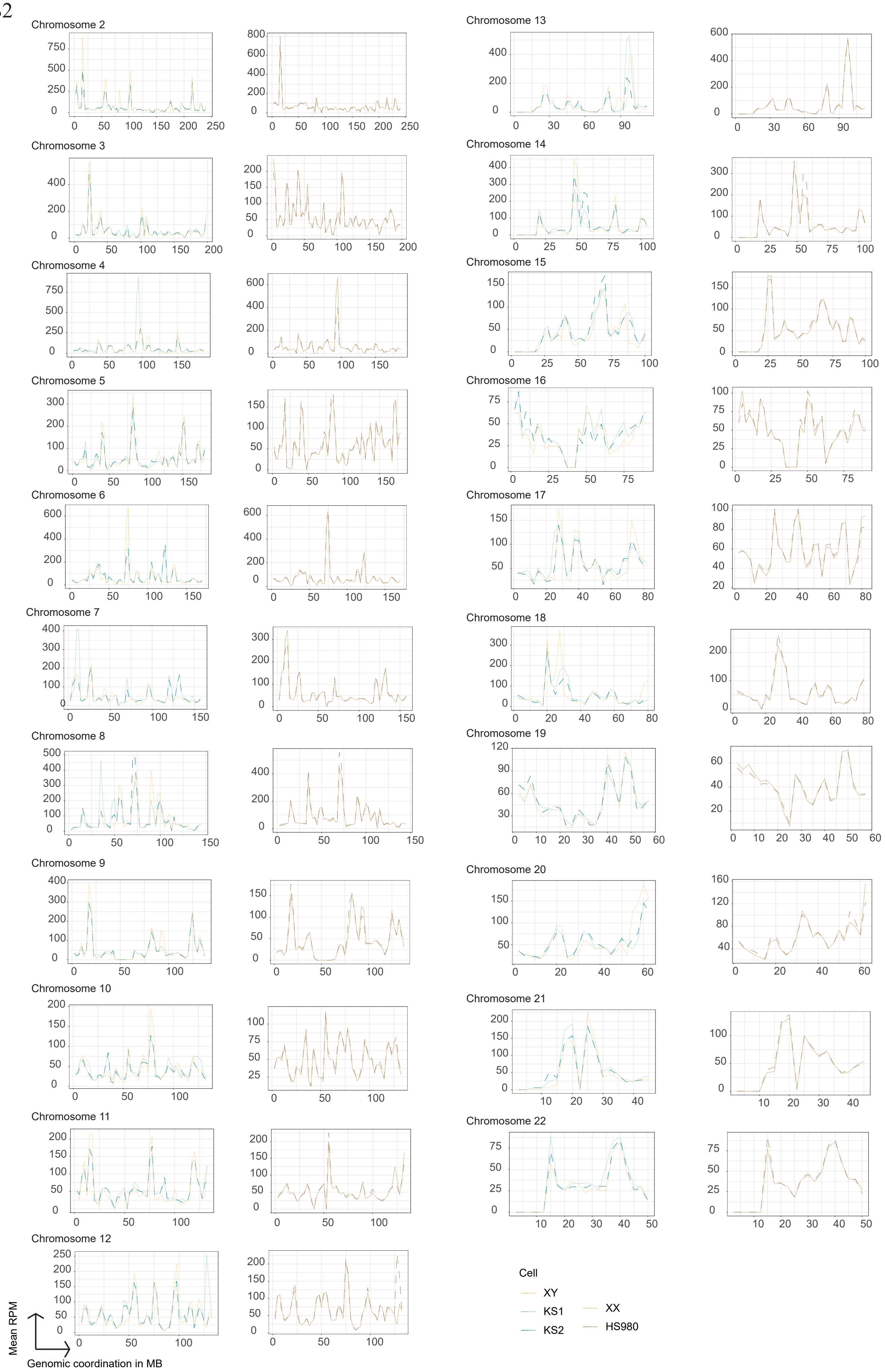


Figure S2. Autosomal gene expressions on genomic coordination, Related to Figure 1.

Figure S3

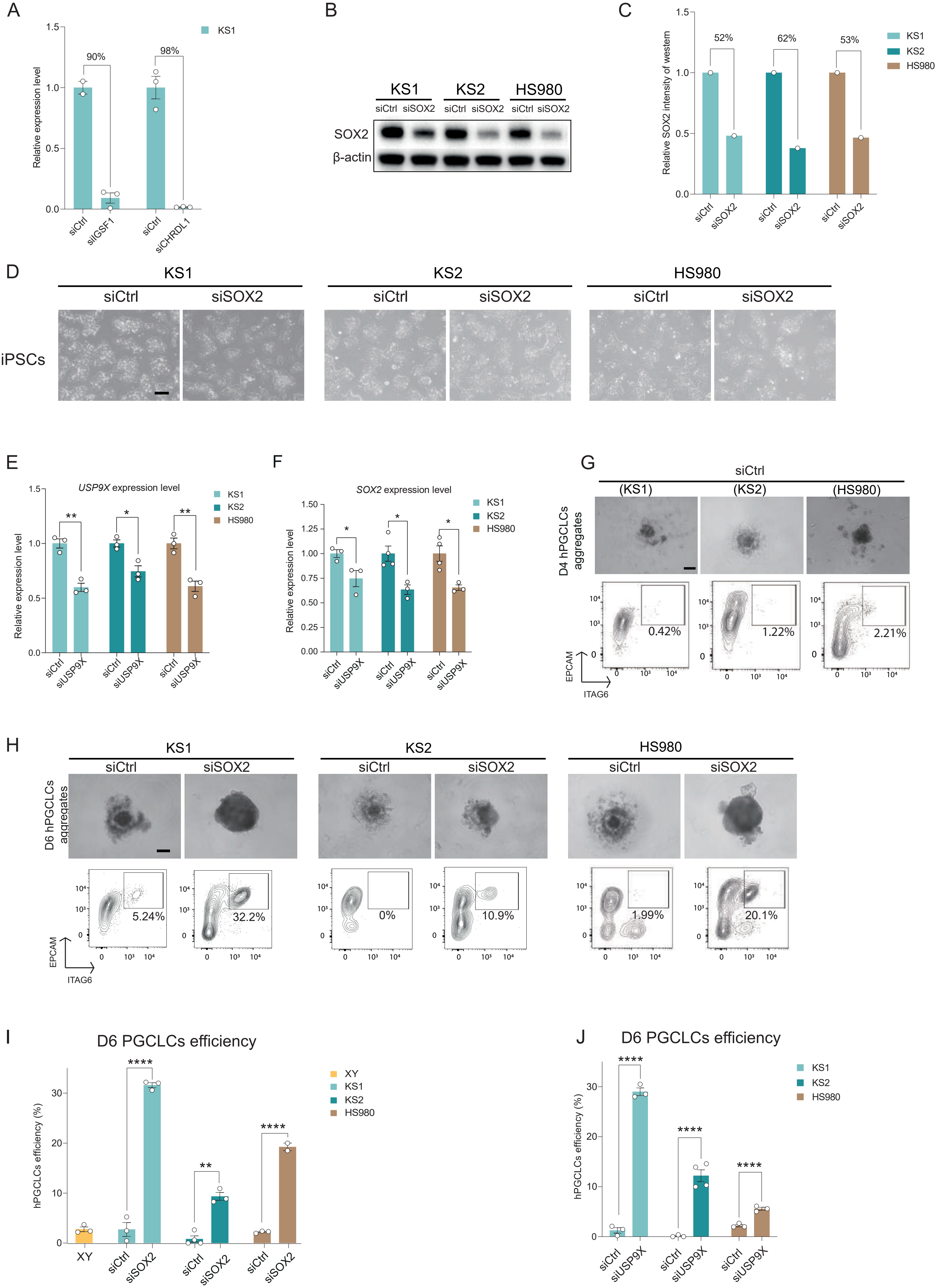
A
KS1
Relative expression level
90%
98%
siCtrl
siIGSF1
siCtrl
siCHRDL1
B
KS1
KS2
HS980
siCtrl siSOX2 siCtrl siSOX2 siCtrl siSOX2
SOX2
β-actin
C
Relative SOX2 intensity of western
52%
62%
53%
KS1
KS2
HS980
D
iPSCs
E
USP9X expression level
Relative expression level
siUSP9X
F
SOX2 expression level
G
D4 hPGCLCs aggregates
(KS1)
(KS2)
(HS980)
0.42%
1.22%
2.21%
EPCAM
ITAG6
H
D6 hPGCLCs aggregates
5.24%
32.2%
0%
10.9%
1.99%
20.1%
I
D6 PGCLCs efficiency
hPGCLCs efficiency (%)
XY
J
D6 PGCLCs efficiency

Figure S3. Knockdown efficiencies, Related to Figure 3.
(A) Knockdown efficiency of *IGSF1* and *CHRDL1* in KS1 iPSCs.
(B) Western blotting showing the decrease of SOX2 protein levels in KS1, KS2, and HS980 after SOX2 KD.
(C) Quantitative analysis of western blotting in B.
(D) Representative image of the PSC morphology of KS1, KS2, and HS980 after 72 hours of SOX2 knockdown. Scale bar is 200 µm.
(E) USP9X relative expression level after *USP9X* KD by real-time qPCR.
(F) SOX2 relative expression level after *USP9X* KD by real-time qPCR.
(G) D4 hPGCLC spheroid morphology and FACS identification of hPGCLC after siCtrl in two KS and HS980 lines. Scale bar, 200 µm. Related to Figure 3L.
(H) D6 hPGCLC spheroid and FACS identification of hPGCLC after SOX2 KD in KS1, KS2 and HS980, respectively. Scale bar, 200 µm.
(I) Statistics of D6 hPGCLC efficiencies after SOX2 KD in KS1, KS2 and HS980 from three independent experiments.
(J) Statistics of D6 hPGCLC efficiencies after USP9X KD in KS1, KS2 and HS980 from three independent experiments.
Data in (A), (E), (F), (I), (J) are shown as mean ± SEM. n.s., non-significant; *$p < 0.05$; **$p < 0.01$; ****$p < 0.0001$ by t test.

Figure S4

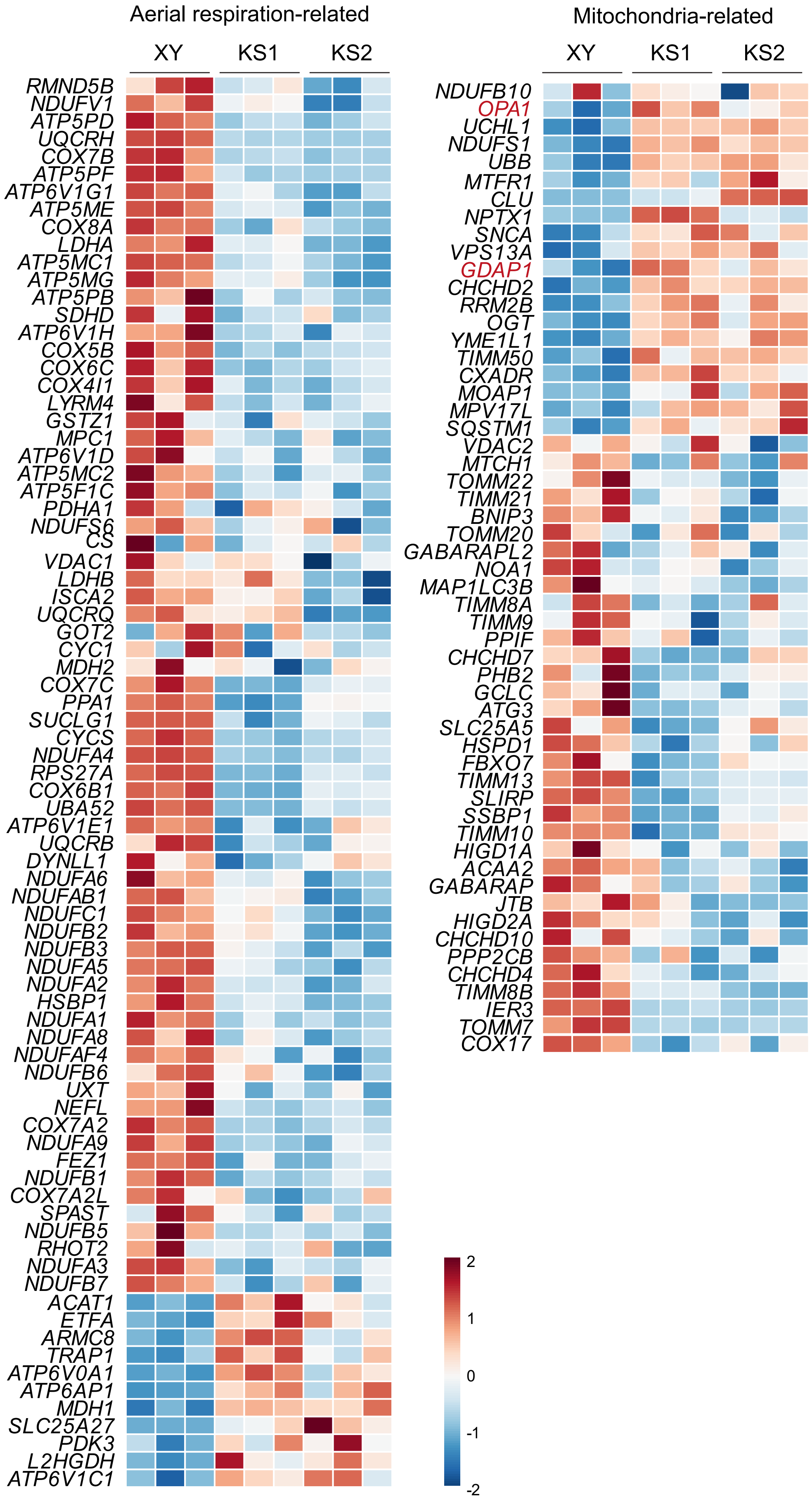


Figure S4. Heatmap showing the KS common DEGs related with mitochondria and aerobic respiration pathways, Related to Figure 4.

Figure S5

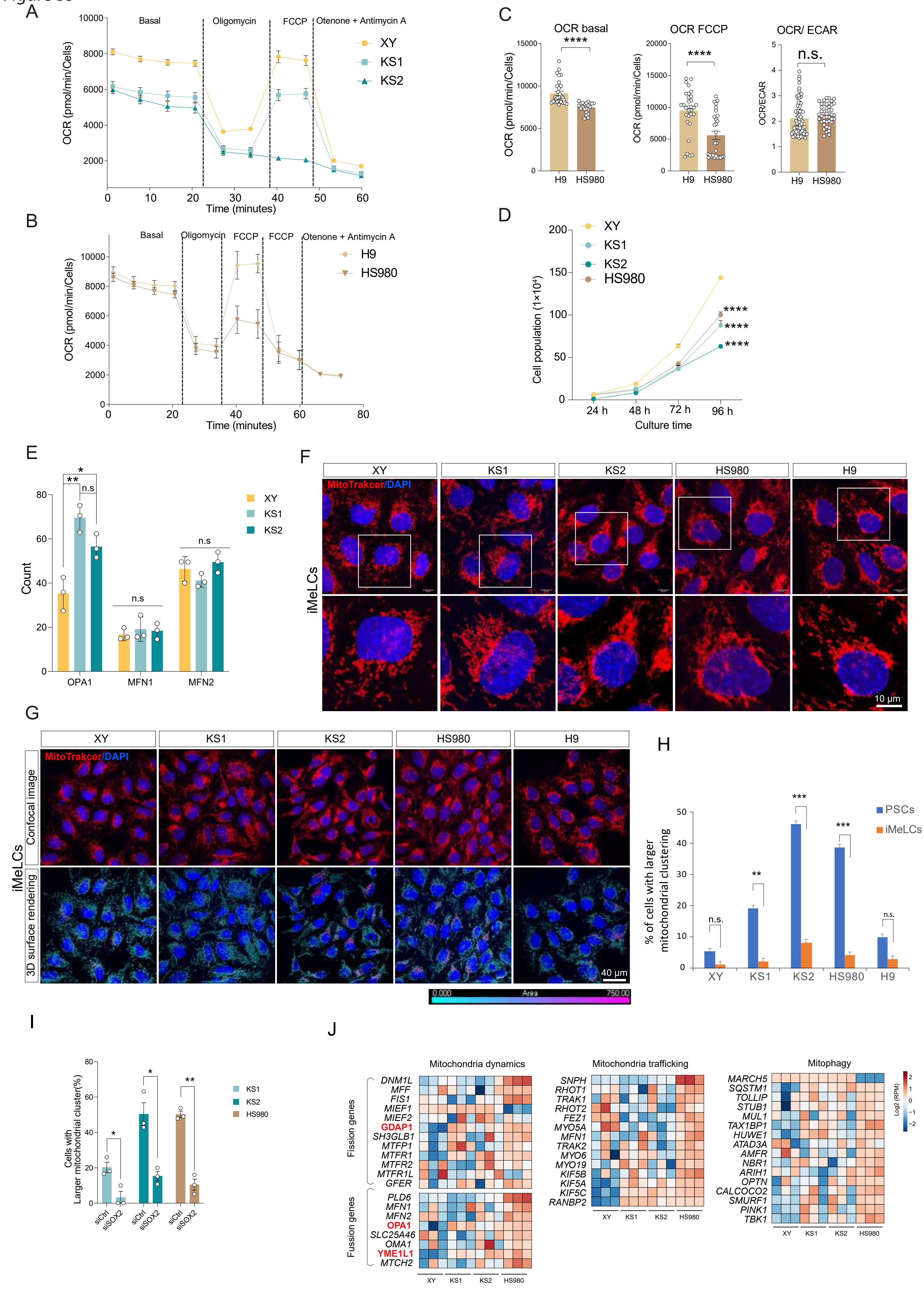
A
Basal
Oligomycin
FCCP
Otenone + Antimycin A
OCR (pmol/min/Cells)
Time (minutes)
XY
KS1
KS2
B
Basal
Oligomycin
FCCP
FCCP
Otenone + Antimycin A
OCR (pmol/min/Cells)
Time (minutes)
H9
HS980
C
OCR basal
OCR FCCP
OCR/ ECAR
****
****
n.s.
OCR (pmol/min/Cells)
OCR/ECAR
H9
HS980
D
XY
KS1
KS2
HS980
Cell population (1×10⁴)
24 h
48 h
72 h
96 h
Culture time
E
Count
OPA1
MFN1
MFN2
n.s
XY
KS1
KS2
F
XY
KS1
KS2
HS980
H9
MitoTrakcer/DAPI
iMeLCs
10 μm
G
XY
KS1
KS2
HS980
H9
MitoTrakcer/DAPI
Confocal image
3D surface rendering
iMeLCs
40 μm
0.000
Area
750.00
H
PSCs
iMeLCs
% of cells with larger mitochondrial clustering
n.s.
XY
KS1
KS2
HS980
H9
I
Cells with Larger mitochondrial cluster(%)
siCtrl
siSOX2
KS1
KS2
HS980
J
Mitochondria dynamics
Mitochondria trafficking
Mitophagy
Fission genes
Fusion genes
DNM1L
MFF
FIS1
MIEF1
MIEF2
GDAP1
SH3GLB1
MTFP1
MTFR1
MTFR2
MTFR1L
GFER
PLD6
MFN1
MFN2
OPA1
SLC25A46
OMA1
YME1L1
MTCH2
SNPH
RHOT1
TRAK1
RHOT2
FEZ1
MYO5A
MFN1
TRAK2
MYO6
MYO19
KIF5B
KIF5A
KIF5C
RANBP2
MARCH5
SQSTM1
TOLLIP
STUB1
MUL1
TAX1BP1
HUWE1
ATAD3A
AMFR
NBR1
ARIH1
OPTN
CALCOCO2
SMURF1
PINK1
TBK1
XY
KS1
KS2
HS980
Log2 (RPM)

Figure S5. SOX2 reduces cellular oxidative phosphorylation and influences mitochondria morphology, Related to Figure 4.
(A) Seahorse data of the XY, KS1, and KS2 PSCs.
(B) Seahorse data of the HS980, and H9 PSCs.
(C) Statistical analysis OCR of basal and FCCP in B.
(D) Growth curve of the PSCs of XY, KS1, KS2, and HS980.
(E) OPA1, MFN1, MFN2 expression level analysis of XY, KS1, KS2 iPSCs from bulk RNA sequenced data.
(F) Mitochondrial morphology of the iMeLCs of XY, KS1, KS2 and HS980 iMeLCs from confocal microscope.
(G) 3D mitochondrial morphology and large cluster in the iMeLCs of XY, KS1, KS2 and HS980 from Imaris software analysis.
(H) Percentage of cells with mitochondria clusters in the iMeLCs XY, KS1, KS2 and HS980. Cluster is designated as surface area> 500 $\mu m^2$.
(I) Percentage of cells with mitochondria clusters in the iPSCs XY, KS1, and KS2 after SOX2 KD. Cluster is designated as surface area> 500 $\mu m^2$.
(J) Heatmaps showing the expressions of selected mitochondria related genes.
Data in (C), (E), (H), (I) are shown as mean ± SEM. n.s., non-significant; $*p < 0.05$; $**p < 0.01$; $****p < 0.0001$ by t test.
Data in (D) are shown as mean ± SEM. $****p < 0.0001$ by two-way ANOVA comparison.

Figure S6

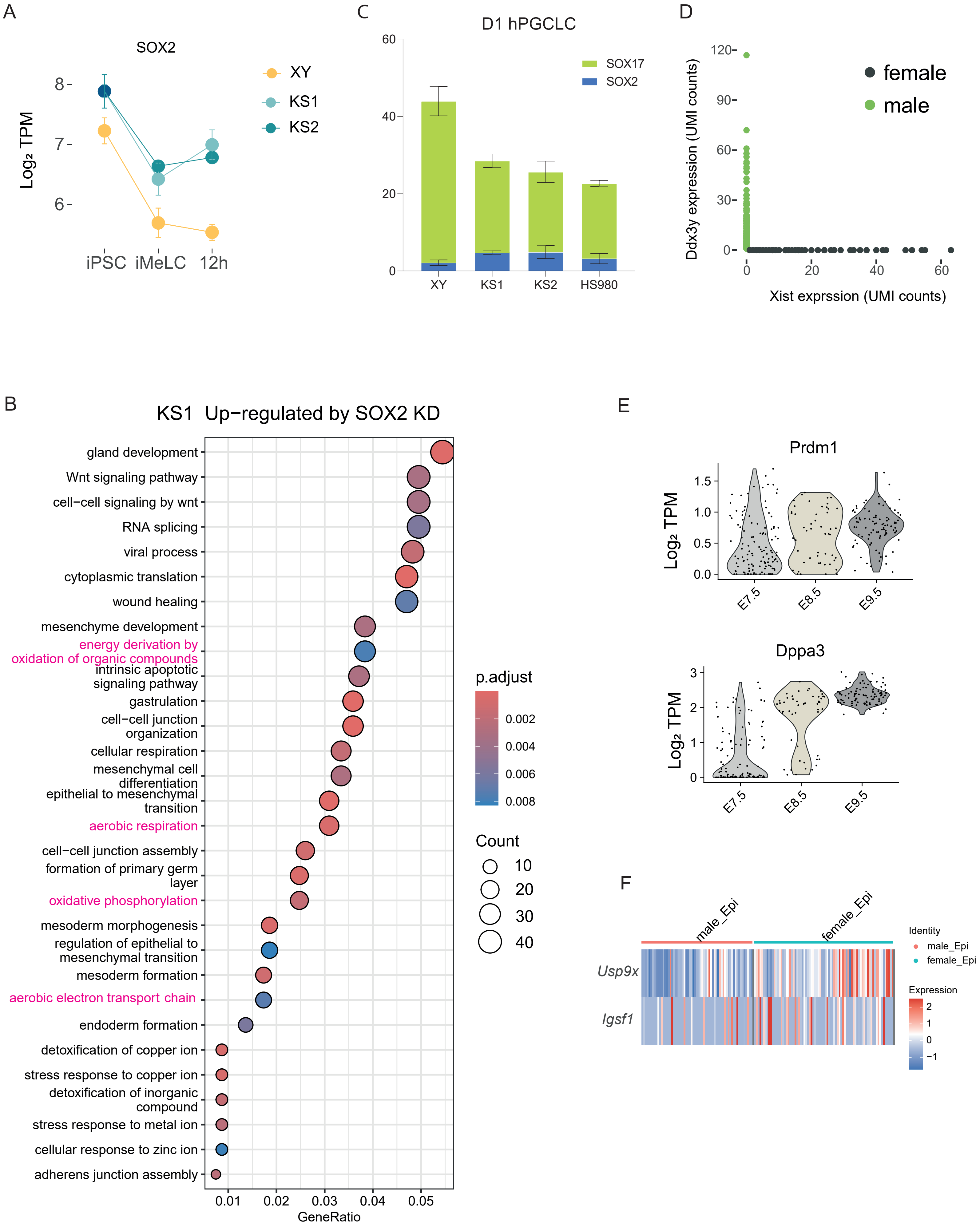

A
SOX2
Log₂ TPM
XY
KS1
KS2
iPSC iMeLC 12h
C
D1 hPGCLC
SOX17
SOX2
XY KS1 KS2 HS980
D
female
male
Ddx3y expression (UMI counts)
Xist exprssion (UMI counts)
B
KS1 Up−regulated by SOX2 KD
gland development
Wnt signaling pathway
cell−cell signaling by wnt
RNA splicing
viral process
cytoplasmic translation
wound healing
mesenchyme development
energy derivation by oxidation of organic compounds
intrinsic apoptotic signaling pathway
gastrulation
cell−cell junction organization
cellular respiration
mesenchymal cell differentiation
epithelial to mesenchymal transition
aerobic respiration
cell−cell junction assembly
formation of primary germ layer
oxidative phosphorylation
mesoderm morphogenesis
regulation of epithelial to mesenchymal transition
mesoderm formation
aerobic electron transport chain
endoderm formation
detoxification of copper ion
stress response to copper ion
detoxification of inorganic compound
stress response to metal ion
cellular response to zinc ion
adherens junction assembly
GeneRatio
p.adjust
Count
E
Prdm1
Dppa3
Log₂ TPM
E7.5 E8.5 E9.5
F
male_Epi
female_Epi
Usp9x
Igsf1
Identity
male_Epi
female_Epi
Expression

Figure S6. mPGC sex related and PGC marker gene expressions, Related to Figure 5 and 6.
(A) SOX2 gene expression changes in the iMeLC and 12h post BMP induction stages in KS1, KS2 and XY.
(B) Up regulated pathways after SOX2 KD in the 12h stage of KS1.
(C) Statistics of SOX2- and SOX17-expressing cell in D4 hPGCLC spheroids of XY, KS1, KS2, and HS980.
(D) Exclusive gene expressions of *Xist* and *Ddx3y* showing female and male PGCs.
(E) Expression of *Prdm1* and *Dppa3* in mPGCs.
(F) Gene expressions of Usp9x and Igsf1 in mouse EpiSCs.

SUPPLEMENTAL INFORMATION

KEY RESOURCES TABLE

| REAGENT or RESOURCE | SOURCE | IDENTIFIER |
|---|---|---|
| Antibodies | | |
| Human/Mouse/Rat SOX2 Antibody | R&D systems | MAB2018 |
| Human NAONG antibody | Miltenyi Biotec | 130-104-732 |
| Alexa Fluor® 488 anti-human CD326 (EpCAM) antibody | Biolegend | 324210 |
| Brilliant Violet 421™ anti-human/mouse CD49f (Integrin-a6 or ITGA6) Antibody | Biolegend, | 313624 |
| Chemicals, peptides, and recombinant proteins | | |
| TRI Reagent | Sigma | T9424 |
| Vitronectin (VTN-N) Recombinant Human Protein, Truncated | Fisher Scientific | A14700 |
| Human plasma fibronectin purified protein | Millipore Sigma | FC010 |
| Recombinant Human BMP-4 Protein | R&D Systems | 314-BP |
| Recombinant Human EGF Protein | R&D Systems | 236-EG |
| Recombinant Human SCF | PeproTech | 300-07 |
| Human Recombinant LIF | STEMCELL Technologies | 78055 |
| Y-27632 (Dihydrochloride) | STEMCELL Technologies | 72307 |
| Animal-Free Recombinant Human/Murine/Rat Activin A | PeproTech | AF-120-14E |
| Knockout™ Serum Replacement | Gibco | 10828-028 |
| NutriStem® hPSC XF Medium | Sartorius | 05-100-1A |
| RPMI 1640 Medium | Sigma-Aldrich | R8758 |
| Glasgow's MEM (GMEM) | Thermo Fisher Scientific | 11710035 |
| 2-Mercaptoethanol | Thermo Scientific | 21985023 |
| Sodium pyruvate | Thermo Fisher Scientific | 11360–070 |
| TrypLE™ Select Enzyme (1X), no phenol red | Thermo Scientific | 12563029 |
| MEM α, nucleosides, GlutaMAX™ Supplemen | Thermo Scientific | 32571036 |
| DPBS, no calcium, no magnesium | Thermo Fisher Scientific | 14190094 |
| Poly-D-lysine hydrobromide | Sigma | P6407 |
| Doxycycline hyclate | Merck | 33429 |
| Critical commercial assays | | |

| | | |
|---|---|---|
| Lipofectamine RNAiMAX Transfection Reagent | Fisher Scientific | 13-778-075 |
| Applied Biosystems Power UP SYBR Green PCR Master Mix | Fisher Scientific | 15390929 |
| 5X All-In-One RT MasterMix | ABM | G490 |
| QIAGEN Plasmid Plus Midi Kit | Qiagen | 12943 |
| Experimental models: Cell lines | | |
| HS980 | Rodin et al., (2014) | N/A |
| XY | Panula, S., et al. (2019) | N/A |
| KS1 | Panula, S., et al. (2019) | N/A |
| KS2 | Panula, S., et al. (2019) | N/A |
| Oligonucleotides | | |
| Control siRNA | | |
| ON-TARGETplus Human SOX2 (6657) siRNA - SMARTpool | Horizon Discovery | L-011778-00-0005 |
| USP9X siRNA | ThermoFisher Scientific | 105099 |
| CHRDL1 siRNA | ThermoFisher Scientific | S534487/s40796 |
| IGSF1 siRNA | ThermoFisher Scientific | s535148/s230594 |
| | | |
| Recombinant DNA | | |
| | | |
| Software and algorithms | | |
| | | |
